# A pre-implementation examination of barriers and facilitators of an electronic prospective surveillance model for cancer rehabilitation: a qualitative study

**DOI:** 10.1186/s12913-023-10445-3

**Published:** 2024-01-04

**Authors:** Christian J. Lopez, Jennifer M. Jones, Kristin L. Campbell, Jackie L. Bender, Gillian Strudwick, David M. Langelier, Tony Reiman, Jonathan Greenland, Sarah E. Neil-Sztramko

**Affiliations:** 1 Department of Supportive Care, Princess Margaret Cancer Centre, University Health Network, 200 Elizabeth St, Toronto, ON M5G 2C4 Canada; 2 Institute of Medical Science, University of Toronto, Toronto, Canada; 3 Department of Physical Therapy, University of British Columbia, Vancouver, Canada; 4 Institute of Health Policy, Management and Evaluation, University of Toronto, Toronto, Canada; 5 Department of Oncology, Saint John Regional Hospital, Saint John, Canada; 6 Department of Medicine, Dalhousie University, Halifax, Canada; 7 Faculty of Medicine, Memorial University of Newfoundland, St John’s, Canada; 8 Dr. H. Bliss Murphy Cancer Centre, Eastern Health, St. John’s, Canada; 9 Faculty of Health Sciences, McMaster University, Hamilton, Canada; 10 National Collaborating Centre for Methods and Tools, McMaster University, Hamilton, Canada

**Keywords:** Implementation, Rehabilitation, Cancer survivorship, Cancer care delivery, Prospective surveillance model, Patient-reported outcomes, eHealth, CFIR

## Abstract

**Background:**

An electronic Prospective Surveillance Model (ePSM) uses patient-reported outcomes to monitor symptoms along the cancer pathway for timely identification and treatment. Randomized controlled trials show that ePSMs can effectively manage treatment-related adverse effects. However, an understanding of optimal approaches for implementing these systems into routine cancer care is limited. This study aimed to identify barriers and facilitators prior to the implementation of an ePSM to inform the selection of implementation strategies.

**Methods:**

A qualitative study using virtual focus groups and individual interviews was conducted with cancer survivors, oncology healthcare providers, and clinic leadership across four cancer centres in Canada. The Consolidated Framework for Implementation Research (CFIR) guided the interviews and analysis of barriers and facilitators based on five domains (intervention characteristics, individual characteristics, inner setting, outer setting, and process).

**Results:**

We conducted 13 focus groups and nine individual interviews with 13 patient participants and 56 clinic staff. Of the 39 CFIR constructs, 18 were identified as relevant determinants to the implementation. The adaptability, relative advantage, and complexity of an ePSM emerged as key intervention-level factors that could influence implementation. Knowledge of the system was important at the individual level. Within the inner setting, major determinants were the potential fit of an ePSM with clinical workflows (compatibility) and the resources that could be dedicated to the implementation effort (readiness for implementation). In the outer setting, meeting the needs of patients and the availability of rehabilitation supports were key determinants. Engaging various stakeholders was critical at the process level.

**Conclusions:**

Improving the implementation of ePSMs in routine cancer care has the potential to facilitate early identification and management of treatment-related adverse effects, thereby improving quality of life. This study provides insight into important factors that may influence the implementation of an ePSM, which can be used to select appropriate implementation strategies to address these factors.

**Supplementary Information:**

The online version contains supplementary material available at 10.1186/s12913-023-10445-3.

## Background

People with cancer endure physical and functional challenges during and after cancer treatment [1, 2] which can significantly affect their quality of life [3]. Treatment-related adverse effects are common and may include fatigue, pain, deconditioning, cognitive changes, and changes to sexual function [4]. However, despite multiple reports of high rates of adverse effects in cancer survivors globally [5–8] and scientific evidence that cancer rehabilitation interventions can mitigate many of these negative outcomes, most cancer survivors do not receive adequate support to manage these challenges [1, 5–8]. Cancer survivors have reported beliefs that these impairments are normal and expected, beliefs that there was nothing that could be done, or not wanting to ask their healthcare provider (HCP) [9].

A Prospective Surveillance Model (PSM) for cancer rehabilitation has been identified as an effective patient-centred, and potentially cost-effective solution to identify and meet the needs of this population [10, 11]. Prospective surveillance facilitates early identification and intervention to manage anticipated treatment-related adverse effects through the routine assessment of cancer survivors’ needs and function across the cancer care continuum [10, 11]. This can be achieved electronically (i.e., an electronic PSM (ePSM). An ePSM is a system that uses electronic patient-reported outcomes (ePROs) to monitor and assess symptoms along the cancer pathway, identify patients’ needs, provide tailored resources to patients, and assist the oncology team in making appropriate and timely referrals to rehabilitation [10, 11].

**Fig. 1:**
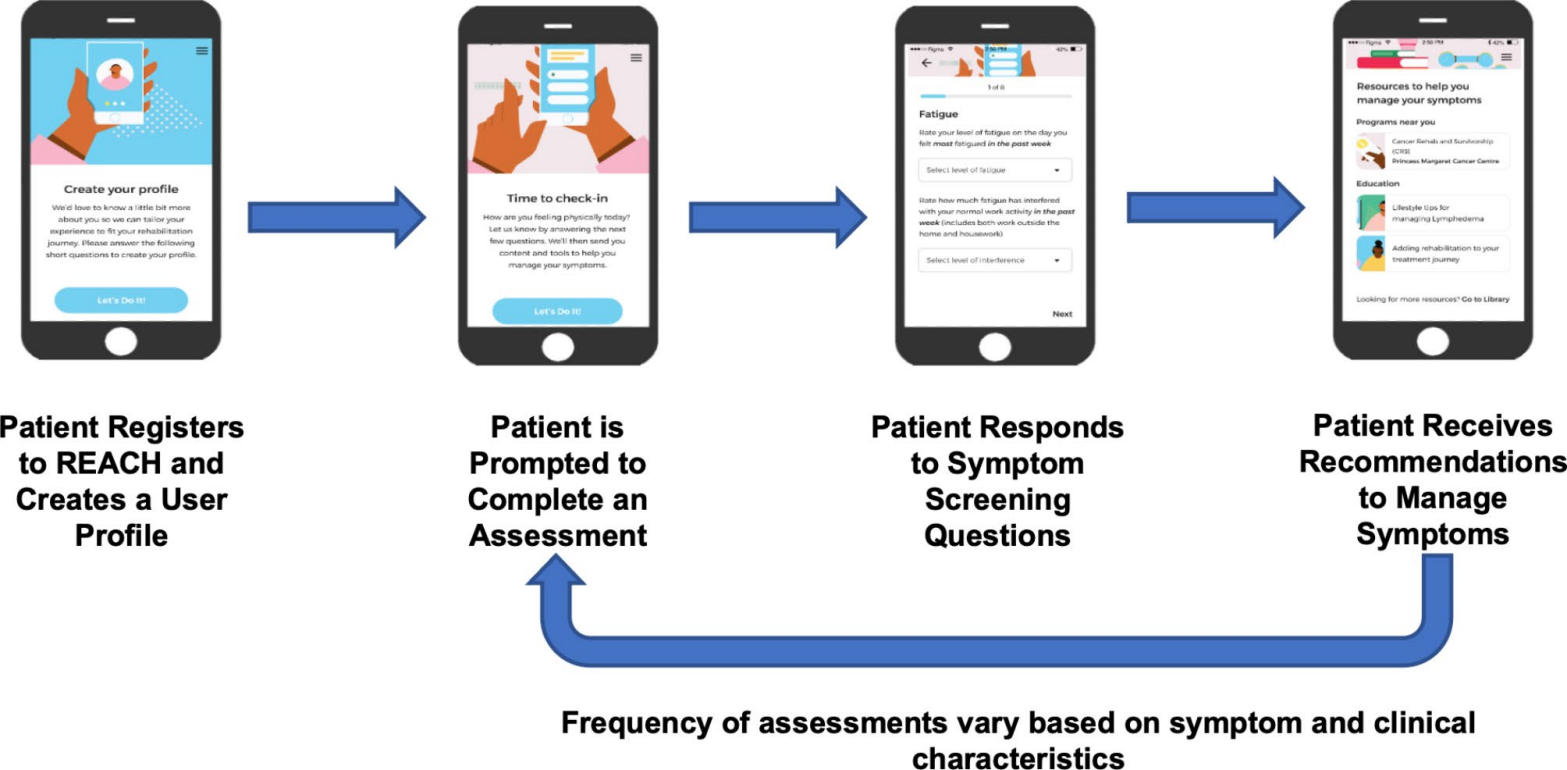
Overview of the REACH system

Randomized controlled trials have demonstrated that ePSMs are effective at improving physical function, symptom management, quality of life, emergency room and hospitalization rates, and overall survival amongst oncology patients, which supports the need for translation from research into practice [12–16]. Given this evidence and as part of a larger program of research, our team developed REACH, an ePSM system designed to remotely screen for and identify adverse effects of cancer and its treatments (such as cancer-related fatigue, difficulties with activities of daily living, and pain) and connect patients to rehabilitation resources based on need (Fig. 1). REACH was developed to be implemented within the clinical setting of four cancer types (i.e., breast, colorectal, head and neck, or lymphoma) through a four-step person-centred design process [17] that included co-design workshops and usability testing with the project’s Patient and Family Advisory Committee (PFAC).

Despite the evidence on the efficacy of ePSMs, less is known about optimal approaches to implementation into routine cancer care [18]. The need to leverage implementation science in cancer care delivery is becoming increasingly recognized to understand and accelerate the integration of evidence-based practices into clinical settings [19, 20]. We previously conducted a scoping review to identify determinants to implementation and strategies employed in studies evaluating the use of an ePSM in cancer [21]. The review provided a foundation for planning the implementation of an ePSM, including REACH; however, implementation of evidence-based practices is highly dependent on local context including organizational features such as culture, leadership, and resources [22]. Furthermore, mapping barriers and facilitators to specific implementation strategies has been suggested to reduce the impact of barriers [23] and tailoring implementation strategies to a given context can increase implementation success [24, 25]. As such, we aimed to build on our scoping review and identify implementation barriers and facilitators that can be used to select and tailor implementation strategies for an ePSM, REACH.

## Methods

### Study design

This qualitative descriptive study [26] was conducted as part of a pre-implementation assessment prior to the implementation of REACH in four Canadian centres. To guide the implementation of REACH into routine clinical practice, we followed a step-by-step theory-informed approach guided by the Knowledge-to-Action (KTA) cycle [27]. The KTA cycle conceptualizes the relationship between knowledge creation and action with activities that may be needed for knowledge application [28]. This study addresses two steps in the KTA cycle, specifically (1) adapt knowledge to the local context and (2) assess barriers and facilitators to knowledge use, to address the next step in this process (i.e., select and tailor implementation strategies). This project was assessed by the Quality Improvement Review Committee at the University Health Network and was issued a formal waiver exempting the study from the requirement for Research Ethics Board approval. Reporting was aligned with the Standards for Reporting Qualitative Research (SRQR) guidelines [29].

### Settings

This study was conducted virtually, with individuals at four Canadian centres: Princess Margaret Cancer Centre (Toronto, Ontario), BC Cancer – Vancouver site (Vancouver, British Columbia), Saint John Regional Hospital (Saint John, New Brunswick), and Dr. H. Bliss Murphy Cancer Centre (St. John’s, Newfoundland). All four cancer centres operate under provincially led single-payer, universal health care systems. Princess Margaret Cancer Centre and BC Cancer – Vancouver are in large, urban centres with relatively high volumes of patients and clinical staff. These centres typically deliver more specialized cancer clinics embedded within their centre, where oncologists and nursing staff will typically exclusively treat one of the four types of cancers REACH is being implemented for. As such, disease sites within these centres typically have their own leadership team (e.g., nurse manager, radiation therapy team lead, physician site lead). In contrast, Dr. H. Bliss Murphy Cancer Centre and the oncology clinic at Saint John Regional Hospital are in smaller urban centres and embedded within general hospitals. These cancer centres typically deliver more general oncology clinics, where oncologists and nursing staff treat more than one of the four types of cancers for which REACH is being implemented. Further, these centres deliver care to many rural and remote patients in their surrounding regions.

### Participants and recruitment

Eligible patient participants included cancer survivors from the research study’s PFAC, which includes representatives from each site and cancer type that will be the focus of the initial implementation of REACH (i.e., breast, colorectal, head and neck, and lymphoma). Cancer survivors from the research program’s PFAC were purposively sampled to obtain perspectives from patients from each implementation site. The PFAC has played a key advisory role as an overarching committee guiding the program of research and has thus far ensured that cancer survivor perspectives remain at the forefront of this project. All patient participants were invited to participate in a focus group with members of the research team and were verbally informed that the meeting would be recorded and only shared with members of the research team. All patient participants verbally consented to have the focus group recorded to facilitate the direction of implementation activities.

Eligible staff participants included HCPs and clinic leadership within each site and within the clinical setting of each of the four cancer types that will be the focus of the initial implementation of REACH. Clinic leads were chosen due to their input and oversight over clinic operations, which would be critical to the success of adopting new interventions. Appropriate HCPs and clinic leadership were identified by the research team leads at each geographical site clinical setting. Potential staff participants were informed of the objectives of the REACH system and the purpose of the interview. Following the first round of interviews, snowball sampling [30] was used to identify additional participants, using suggestions from participants as to other individuals who may provide important perspectives for the implementation of REACH in their respective settings.

### Data collection

Qualitative data were collected through focus groups between October 2020 and April 2022. In select situations, key individuals were unable to accommodate focus group schedules; thus, individual interviews were conducted. One week prior to each focus group and interview, participants were provided with a brief video explaining the purpose of REACH and demonstrating the initial design of the system to set the stage for the conversation.

All focus groups and interviews began with a 5-minute presentation to provide further details not included in the pre-meeting video (e.g., symptoms being screened, frequency of screening, and types of resources provided to patients). A semi-structured focus group and interview guide, informed by the Consolidated Framework for Implementation Research (CFIR) [31] was used to elicit an understanding of potential barriers and facilitators to implementing REACH within each clinical setting (see Additional File 1 and Additional File 2 for the patient participant and staff participant focus group guides, respectively). The CFIR is a widely used determinant framework in implementation science [32] that includes 39 constructs within five domains (characteristics of the intervention, inner setting, outer setting, characteristics of individuals, and the process of implementation) known to influence implementation outcomes and success [31]. Focus groups were conducted virtually via the Zoom platform. Three members of the research team trained in qualitative interviewing and implementation science (CL, JMJ, and SNS) conducted all the focus groups. In cases where power imbalances were evident, particularly during focus groups with clinic staff, the facilitators used appropriate techniques such as redirecting questions or encouraging less vocal participants to share their thoughts. All participants were assured that their responses would be anonymized and kept confidential. All focus group and interview data were recorded and transcribed verbatim.

### Data analysis

Qualitative analysis was performed by two members of the team (CL and SNS) using Dedoose software. A thematic analysis was conducted using a hybrid deductive and inductive approach [33]. First, each recording was carefully listened to before and after transcription, and each transcript was read and re-read to become familiar with the interview and ensure the focus group was reproduced correctly. The CFIR codebook template with pre-populated definitions and coding guidelines was used to facilitate the analysis in Dedoose [34]. The code descriptions were adapted to reflect the use of the CFIR for an ePSM. To ensure the quality and consistency of the coding, four focus groups were selected to be coded by both coders independently, and both coders then met to clarify aspects of the codebook. Each remaining transcript underwent a process of deductive coding by one independent coder and double-checked by a second; both coders continued to meet to provide an opportunity to discuss any varied interpretations of the codes and ensure fragments of information from the focus groups were provided with the appropriate codes. Following deductive coding, the coded data within each CFIR construct were compared to ensure consistency, and similarities and differences between these references informed any adjustments to the code description and the included data. Following an inductive approach, data within each coded construct were categorized into themes as appropriate. Descriptions of each theme were created, and representative quotes for each construct were chosen.

## Results

### Participant characteristics

A total of 13 focus groups and nine individual interviews were conducted. This included one focus group with all 13 patient participants from the study’s PFAC, with the rest of the focus groups and individual interviews conducted with a total of 56 clinic staff (Table 1). As part of the formal waiver exempting this study from the requirement for Research Ethics Board approval, patient participant demographics were not collected. However, the 13 members of the study’s PFAC represent individuals from each of the study’s four sites and cancer types. The patient participant focus group lasted approximately 2 h. Most of the clinic staff interviewed were from Ontario (n = 33), followed by New Brunswick (n = 9), Newfoundland (n = 7), and British Columbia (n = 7) with various roles, including oncologists, nursing, and allied health, as well as leadership and management personnel, many of which also had an active clinical role or background. An average of four clinic staff participants (range = 2–7 participants) participated in each clinic staff focus group. The average duration for the clinic staff focus groups was 43 min (range = 23–56 min), while the average duration for the individual clinic staff interviews was 35 min (range = 30–50 min).

**Table 1:** Clinic staff primary professional roles

Role		n
**Leadership/Management**		
	Ambulatory Clinic Nurse Manager	4
	Director (clinical, nursing, regional)	3
	Coordinator (patient care, program)	2
	Advanced Practice Nurse Educator	2
	Radiation Therapy Lead	1
**Oncologist**		
	Radiation Oncologist	10
	Medical Oncologist	7
	Hematologist Oncologist	7
	Surgical Oncologist	1
**Nursing**		
	Clinical Nurse Specialist/Coordinator	6
	Registered Nurse	4
	Nurse Practitioner	2
	Nurse Navigator	1
**Allied Health**		
	Speech-Language Pathologist	2
	Occupational Therapist	1
	Physiotherapist	1
	Registered Dietician	1
	Social Worker	1

Of the 39 CFIR constructs, 18 were identified as relevant determinants to the implementation of REACH within each of the four disease site settings (breast, colorectal, head and neck, and lymphoma). A summary of the determinants with representative quotes is presented in Table 2. The detailed results in the context of the five CFIR domains and the most relevant constructs are presented below.

**Table 2:** CFIR barriers and facilitators

CFIR Construct	Barrier or Facilitator	Description	Example Quote
**Intervention Characteristics**			
Adaptability	Barrier	• Difficult to tailor the system to the variability of patients, including the treatment options and durations, languages spoken, and comfort with technology.	“There are so many different [clinical] presentations and so many different scenarios. Even once treatment has started, sometimes it’s chemotherapy, sometimes it’s a pill, sometimes it’s antibody therapy, which doesn’t have a lot of side effects.” [Oncologist]
	Facilitator	• Having a flexible system for patients. This includes the flexibility of when patients can register to the system and the ability to access the system on any electronic device. • The ability to tailor the symptoms addressed to the cancer type and treatment status of the patient. • The ability to offer resources through different modes of delivery (e.g., reading material, videos, online and in-person programs).	“I love the idea of constantly reintroducing it and being able to register at different times because maybe chemo is an easy ride, but when you have a bilateral mastectomy, you may need [REACH]. You never know.” [Patient]
Relative Advantage	Barrier	• Concerns about the possible redundancy of the system with questions and recommendations from health care providers to manage cancer-related impairments. • Concerns about replacing or decreasing the personal contact and discussions with health care providers.	“These kinds of questions for the symptoms do come up within the course of the days and weeks that they’re here with various members of the team because they have a lot of face-to-face contact with clinicians. So, I guess it’s not very clear whether this is going to be an add-on to the additional dialogue that we’ll be having with patients.” [Radiation therapist] “The conversations and dialogue are essential. I’d be concerned that you’re using technology that’s going to create this algorithm for care that diminishes the personal contact for me with my team.” [Patient]
	Facilitator	• Patients receive an immediate recommendation on the system to manage their symptom(s). • Potential improvements in processes for patients to access cancer rehabilitation resources. • Provides patients with a centralized place to access trustworthy information.	“If they get something directly like tips or resources instead of just creating a body of knowledge, so they feel that they’re getting something back for sharing their information, I can sell that part to a patient.” [Clinical nurse coordinator] “[REACH] is almost getting in the way of Doctor Google, and I really applaud that. It’s kind of a personal resource for information, as opposed to falling down that rabbit hole at 3 in the morning on the internet.” [Patient]
Complexity	Barrier	• Concerns about patients’ ability to use the system independently and manage technical challenges or questions patients may have. • Challenges managing concerning symptoms remotely.	*“So just noting that in terms of the onboarding and registration of the patients, we need to make sure we have enough support available to the patients and something in the system design that will help them get through that because it sounds like it may be a bit of a hurdle to do the registration piece if it’s not designed properly.”* [Oncologist] “Trying to pick up the severe dysphagia of the patients that are actually aspirating is extremely important. But since [REACH] is not generating the [symptom data] to clinicians, isn’t there a risk that patients will say, yes, I’m aspirating, and assume that we know about that because they put it down in the app?” [Speech-language pathologist]
	Facilitator	• Ensure patients are aware of the remote nature of the system and that scores are not monitored by a health care provider.	“I do like the idea of having an alert for the patient, stating they had a sudden increase or drop in this symptom or they’re on one extreme end of the scale, so review this with your physician.” [Clinical site lead and oncologist]
Design Quality and Packaging	Facilitator	• The ability for patients to view how their scores compare over time. • The ability to save the resources recommended to view at a later time. • Ensure the resources recommended are up to date.	“I like the option to save that information and not have to go through it all right away. It may be overwhelming if you’re given a bunch of different resources to go through.” [Patient]
Evidence Strength and Quality	Barrier	• Skepticism of the system’s benefits on clinical and health service outcomes. • Skepticism of the validity of the screening questions patients are asked to complete for each symptom.	“My only concern is, what are the resources on REACH telling them and teaching them?” [Oncologist] “I was just wondering about the symptom selection and the validity of the implementation. Is this the first time this is going to be piloted essentially?” [Speech-language pathologist]
**Outer Setting**			
Patient Needs and Resources	Facilitator	• The potential to fill gaps in care by providing patients with resources and supports to manage their symptoms.	“Patients can start to feel that disconnection with the cancer center when they’re finishing treatment, and even insecure because they’re going to be back to their regular environment without the support of all the team. So I think it’s really a great project.” [Clinical site lead and oncologist]
Cosmopolitanism	Barrier	• Concerns about the limited number of rehabilitation services and their capacities to respond to impairments identified by the system.	“The other challenge we have in Newfoundland is we don’t have the portfolio of services that some of the bigger places have to address these issues.” [Nurse practitioner]
	Facilitator	• The potential to build local connections between the cancer centre and community programs and services.	“This project might be able to connect what we have already and to build bridges here locally.” [Oncologist]
External Policy and Incentives	Facilitator	• Ensuring institutional departments and teams such as privacy, security, and legal are engaged and that the system meets all necessary standards.	“We have to consider administrative pieces such as whether this information should somehow be part of [a patient’s] medical record, or whether [clinicians] are going be able to access the information patients enter. Or whether there are issues we have to deal with from a privacy and security perspective for us to implement this.” [Clinical site lead]
**Inner Setting**			
Structural Characteristics	Barrier	• Centers where disease site clinics (e.g., breast, lymphoma) or disciplines (e.g., surgical oncology and medical oncology) are dispersed or located in different settings, may require more time or effort to implement the system due to having different work flows to consider and more staff to engage. • Patients may be receiving treatment (e.g., surgery) at additional sites outside the cancer centre and therefore may have fewer opportunities to learn about the system.	“I just want to point out that here [in the centre], the haematology team is currently outside the cancer program. The haematology team doesn’t use the same area as the other cancer sites and they’re not under the same [organizational] structure.” [Oncologist]
Implementation Climate *(sub-constructs compatibility and relative priority)*	Barrier	• Concerns about the potential overlap with existing or upcoming electronic patient-reported outcomes systems used in the setting. • Other initiatives and projects may be prioritized over the system by the setting and delay or hinder the implementation of the system.	“There are many applications, and I worry patients might get mixed up. What if we have patients on treatment that are [on REACH], and they’re answering these questions [on REACH], and they don’t answer their questions on another platform? I worry that patients might get confused.” [Oncologist] “I would suggest not to go live right now. The learning curve for [the new EMR] in the clinics is going to be huge. People won’t have the time or bandwidth for anything else.” [Ambulatory clinic nurse manager]
	Facilitator	• Integrating the approach of registering patients on the system into processes used to communicate with patients and to provide patients with educational materials.	“We have processes for providing patients with information. It might be good if we integrate [REACH] into our existing processes. If a patient is going to start treatment, they get a whole package of information about their treatment. If information about this system was in there, then that might be helpful.” [Ambulatory clinic nurse manager]
Readiness for Implementation *(sub-construct available resources)*	Barrier	• Limited time for staff to introduce the system to patients during clinic visits. • Concerns about the ability for the setting to respond to an increase in patient calls or visits as a result of the system.	“If this has any operational impact on our clinical team, then it’s going to be really hard to move forward and take a lot more time [to implement].” [Site director]
**Individual Characteristics**			
Knowledge and Beliefs	Facilitator	• Ensuring patients and staff are familiar with the characteristics of the system and how the system is different from other electronic systems used by patients in the setting.	“It would be nice to give us a refresher on the system before the launch. Even to say, okay, these are the types of questions asked and resources that are going to be available in this app. That’ll just help staff get on board.” [Clinical site lead and oncologist] “It would be important to make sure you draw a distinction between the purpose of this system versus the PROMs that we’re doing because you would potentially have patients who would be offered both. At first, [both systems] are going to seem similar, and they might not understand why they’re signing up for two different things.” [Ambulatory clinic nurse manager]
**Process**			
Engaging *(sub-constructs opinion leaders and key stakeholders)*	Facilitator	• Ensuring clinic leadership and management are engaged and provide approval to implement the system in the setting. • The ability to receive feedback on how the system can be integrated into the clinic workflow and a patient’s cancer pathway. • Ensuring patients and staff are provided with engaging educational material to improve the adoption and uptake of the system. • Ensuring patients are provided with reminders on the system to complete their symptom reporting.	“For each of the disease site groups, there is a site leadership structure, and any project that anybody wants to do has to go through that structure to determine whether it moves forward.” [Oncologist] “The best way to get people to use [REACH] is to clearly and immediately demonstrate that it has a function for them.” [Patient] “I think it’s better [to introduce REACH] after people have the time to process their whole diagnosis and they’re in a better headspace to sort of open their mind to this.” [Patient]

### Intervention characteristics

Adaptability, complexity, and relative advantage emerged as key intervention characteristics that could influence implementation. Design quality and packaging and evidence strength and quality were also discussed (see Table 2).

#### Adaptability

The ability to adapt REACH to fit the needs of patients was an important factor for implementation. Clinic staff highlighted the variability of patients, such as treatment options and durations by cancer type, as a potential challenge. Several oncologists indicated that this variability might make it difficult to offer a symptom screening system with optimal surveillance schedule for all the sub-groups. Other HCPs also indicated that the variability in languages spoken other than English and the level of comfort with technology (for example, by age) could be a potential barrier.

The REACH system’s flexibility was considered an important facilitator, including the ability to tailor symptom-specific questions and resources by cancer type, treatment status (i.e., on active treatment, post-treatment), province and institution. Additionally, the plan to allow patients to register for REACH at any point between diagnosis and two years post-treatment, rather than at a fixed timepoint, may enable more patients to learn about the system and use it when they feel it is appropriate.

#### Relative advantage

Nursing staff and radiation therapists were concerned that depending on the frequency of the symptom reporting, REACH might overlap or conflict with discussions and recommendations on how to manage various symptoms. Patients also raised concerns about the system potentially replacing their personal connections with their oncology care providers.

The perceived advantages of implementing REACH included the possibility of an improved process to access rehabilitation services, particularly for those who are no longer having regular follow-ups at the cancer centre. Advantages could also include providing patients with a centralized place to access trustworthy information and directing them away from internet search engines. Patients and HCPs underscored the conceivable benefit of providing patients with a direct response from the system, as opposed to other routine symptom reporting systems that require clinicians to view and respond to scores during clinic visits; this often did not happen, and patients’ concerns were not always addressed in a timely manner.

#### Complexity

HCPs raised concerns about the potential challenges of using a remote patient-reporting system for patients, such as difficulties using the system independently and managing technical issues. Second, HCPs were concerned about the complexity of managing high scores for symptoms reported on REACH. When asked how to address this concern, HCPs noted that patients should be made aware that their scores were not being actively monitored by their HCP and to include a clear message in the system to contact their provider or clinic if indicated. Additionally, they suggested that REACH can be presented as a patient-directed tool, where patients are prompted to share information from REACH with clinicians.

### Outer setting

With respect to the outer setting, the needs of patients, cosmopolitanism (i.e., the degree to which an organization is networked with other external organizations), and external policies emerged as key factors that could influence implementation.

#### Patient needs and resources

The extent to which REACH would be able to meet the needs of patients receiving care at the centre was seen as an important factor for future implementation. Patients and staff highlighted the potential for REACH to fill gaps patients care, noting that patients often feel abandoned following treatment and are not aware of available resources to manage treatment-related adverse effects. REACH might provide patients with a sense of reassurance and support to manage their symptoms.

#### Cosmopolitanism

Staff raised concerns about the limited number and capacity of programs available. Concerns about capacity were shared across all sites, while concerns about the limited number of resources were primarily voiced by staff working in sites with limited rehabilitation services built into the current clinical care delivery model (i.e., Vancouver, Saint John, and St. John’s). Conversely, clinic leadership highlighted the potential for REACH to improve the connections with rehabilitation services in the community. They indicated that while community programs for cancer rehabilitation may exist, there is limited connection to HCPs at the cancer centre. They noted that REACH might facilitate the development of new or strengthen existing connections by raising staff awareness. Patients highlighted the potential for REACH to improve communication with family physicians about cancer-related impairments post-treatment.

#### External policies and incentives

Clinic leadership highlighted institutional policies that might impact implementation of REACH, such as limitations on email communication with patients (to inform them about REACH). Second, clinic leadership indicated that the ability of patients to register on the system independently and remotely without direct support from the clinic or research staff might be impacted by institutional consent policies. Finally, clinic leadership and HCPs highlighted the importance of ensuring the data storage and security of the REACH system conforms to the institutional policies and practices.

### Inner setting

With respect to the inner setting, the readiness for implementation and implementation climate emerged as key factors that could influence implementation. Structural characteristics were also discussed (see Table 2).

#### Readiness for implementation

Staff at all four sites raised concerns about the limited resources available to implementing REACH. First, clinic leadership and HCPs, including oncologists and nursing staff, were concerned about their ability to respond to an increase in calls or visits to the clinic from patients using REACH who were concerned about a symptom. Second, they were concerned about the lack of time they may have to introduce and explain the REACH system to patients.

#### Implementation climate

Staff raised concerns about the overlap with existing symptom reporting systems, wanting to ensure patients were not being asked to report the same symptoms at similar intervals. While concerns about the overlap were shared across all sites, this was more pronounced in the study’s largest site (Toronto, Ontario). Clinic leadership emphasized the importance of considering other clinic initiatives and priorities (e.g., new electronic medical record systems) when determining when and how to implement REACH. HCPs, including nursing staff and radiation therapists, indicated their ability to introduce the REACH system to patients during a clinic visit would be restricted by additional tasks to complete and information to provide to patients.

When asked how to address these concerns, clinic leadership and HCPs noted that the process for introducing REACH to patients should be integrated within existing communication channels, such as embedding information into educational materials already routinely provided to patients, or sending information about REACH through a patient portal. This would limit staff’s responsibilities, and provide a more reliable process for raising awareness of REACH among many patients.

### Individual characteristics

#### Knowledge and beliefs

Clinic leadership and HCPs highlighted the importance of ensuring patients and clinicians are educated and familiar with REACH, including its purpose, the symptoms being screened, frequency of reporting, and types of resources provided to patients. This was particularly the case for settings where patients might be offered and using other electronic reporting systems throughout their care.

### Process

#### Engaging

Involving staff who hold leadership and management positions was strongly suggested, for example, clinical directors, and nurse or allied health managers, to provide the necessary approvals for implementation. HCPs indicated that involving these individuals would be vital for organizing and preparing for the implementation of the system, including providing feedback on how REACH can be integrated into the clinic workflow and reminding or encouraging staff to promote the system to patients.

Attracting and involving clinic staff in the implementation effort, as well as utilizing effective engagement strategies to promote the system and encourage its sustained use by patients, were also suggested. Clinic leadership and clinicians highlighted specific clinical roles that might be better suited to introduce REACH to patients, such as ambulatory clinic nurses and radiation therapists. HCPs and patients suggested developing engaging materials for REACH, such as videos and handouts that highlight the personal benefit of using REACH, such as the ability to track symptoms over time and receive resources to manage their impairments.

To distribute this information to HCPs, clinic leadership suggested presenting the system and implementation plan during staff meetings, recording presentations to accommodate different schedules, and providing feedback about adoption following launch. Patients and HCPs suggested distributing information to patients at multiple points through electronic systems and physical copies in clinic. However, they both recommended avoiding introducing the system to patients at the time of diagnosis as patients are overwhelmed and are provided with a considerable amount of information. Patients and clinicians suggested reminders within the REACH system to ensure patients complete their questions and use the resources recommended to them.

## Discussion

In this study, we aimed to identify implementation barriers and facilitators that can be used to develop a tailored implementation strategy for an ePSM which will be launched at four regional centres across Canada. Although previous studies have explored the use of an ePSM in routine cancer care, our study explores the perspectives of HCPs, clinic leadership, and patients prior to implementing an ePSM using a well-known implementation science framework (i.e., CFIR) to categorize barriers and facilitators. We describe perspectives from clinic staff and patients on the integration of an ePSM into clinic workflows, methods to engage staff and patients in the implementation effort, and features of an ePSM system that may hinder or enable implementation.

This study builds on an existing body of literature examining the implementation of similar systems and calls for improved guidance on the optimal implementation strategies for these systems [18, 35–37]. The findings from this study reflect previously reported determinants of implementation while also adding to the existing body of evidence in this area of study. For instance, the complexity and relative advantages of the system (intervention characteristics), the compatibility between the ePSM and existing workflows (inner setting) and meeting the needs of patients (outer setting) were commonly raised as potential concerns for implementation. These findings also align with a prior scoping review we conducted on the approach to implementing ePSMs in oncology [21]. However, this study identifies and highlights additional factors to consider, such as the adaptability of the system, the level of services available for patients and connections with community services (i.e., cosmopolitanism), policies for privacy, security, and consent, and engaging and involving various stakeholders throughout the implementation process.

Engaging the deliverers and recipients of an evidence-based intervention is often an overlooked part of implementation [38]. However, engaging relevant stakeholders early and often throughout the implementation process has the potential to ensure the sustainability of evidence-based interventions in healthcare settings [39–41]. In fact, stakeholder engagement has been reported as a strong enabler for implementing an ePSM in an oncology setting [42]. This study also highlights the importance of engaging diverse knowledge users with distinct roles in implementation, such as patients, HCPs, and clinic management to facilitate the implementation of an ePSM. Practical engagement strategies, such as training and educating patients and HCPs, leveraging existing communication channels and processes to distribute educational materials, and integrating into the clinic workflows will all require assistance and involvement from middle managers. These findings align with a prior critical interpretive synthesis on the roles, activities, and impacts of middle managers in facilitating the implementation of evidence-based practices in healthcare settings, including convincing HCPs of the need for and benefit of a project, adjusting the implementation to the organizational context, and assisting with monitoring and evaluating the progress of a project [43].

The engagement of relevant stakeholders is also critical to address key implementation factors identified in this study from the inner setting domain related to the implementation climate and readiness for implementation. This includes the importance of considering the compatibility of the ePSM with clinical workflows and other electronic patient-reporting systems used in the setting and the impact on staff time and clinic resources resulting from the implementation effort. This is consistent with priority recommendations for the implementation of patient-reported outcomes systems in oncology, where factors such as assessing current staff capabilities and service requirements, as well as mapping clinic workflows and processes to enable implementation, were considered the highest priorities [44]. By assessing the clinic workflow and staff capabilities, the findings from this study suggest that integrating the introduction of REACH into patient discussions with HCPs who have a key role in patient education and patient self-management (e.g., ambulatory clinic nurses and radiation therapists) may be critical for the successful implementation of REACH. This is also consistent with findings from a multinational survey which demonstrated that compared with physicians, nurses and allied health professionals were less likely to perceive disruptions in clinical care during the use of patient-reported outcomes [45].

Similar to our findings, a previous scoping review on the role and impact of digital health solutions in oncology supportive care, identified challenges to implementation, including developing systems that suit most patients while also ensuring sufficient adaptability for use in this clinically diverse population [46]. The findings from this study suggest that implementers should prioritize the adaptability of an ePSM by customizing the symptoms being screened based on patient characteristics (e.g., cancer type, treatment status, etc.). Similar to this study, the perceived usefulness of reporting symptoms electronically has been identified as a patient-level determinant of implementation [47]. Therefore, the adaptability of an ePSM may enable implementation by ensuring that the symptoms screened are considered relevant and important to patients and clinicians. Additional adaptable features of an ePSM, such as translations to different languages, may also improve the uptake and use of these systems.

Cancer survivors may continue to experience physical and psychosocial needs related to their cancer experience for many years after treatment [48, 49], and studies evaluating the use of ePSMs in routine cancer care have reported that these systems can enable clinicians to better support patients through referral to appropriate supportive care options [50, 51]. Participants in this study highlighted the importance of considering how the system will meet both patient needs, and the centre’s policies for privacy and security. Ideally, this should be considered early on during the development of the system to avoid significant delays in the implementation effort and provide staff with greater confidence in adopting and promoting the system in their setting. Lastly, participants in this study underscored the importance of considering the number of rehabilitation services available in the community to treat impairments identified by the system. Self-management educational interventions centred on improving patients’ knowledge, skills, and confidence in managing cancer-related impairments have the potential to improve various symptoms and quality of life [52–54]. Therefore, similar to the REACH system presented to participants in this study, implementers should consider developing ePSM systems that direct patients to self-management resources and online programs to address this concern.

The findings from this study provide useful insight to address the next step in the implementation process for REACH guided by the KTA cycle (i.e., select and tailor implementation strategies) [28]. The Expert Recommendations for Implementing Change (ERIC) taxonomy provides 73 possible discrete implementation strategies, and these strategies were categorized by their importance and feasibility to assist in the selection of strategies for a particular setting [55, 56]. Based on the strategies categorized as feasible and important, strategies that may be considered for REACH and similar ePSMs include: (1) organize implementation teams and team meetings (i.e., develop teams of stakeholders and provide protected time for teams to reflect on the implementation effort, share lessons learned, and make refinements to the implementation plan); (2) develop and organize quality monitoring systems (i.e., develop and organize systems and procedures that monitor outcomes for the purpose of quality improvement); (3) distribute educational materials (i.e., develop materials such as handouts and videos to make it easier for stakeholders to learn about the system); (4) provide local technical assistance (e.g., develop a process for patients to receive support for technical issues they encounter while using the system); and (5) tailor strategies (e.g., map the clinic workflow and modify when and how educational materials are distributed to patients based on identified barriers and enablers).

The use of implementation science frameworks to guide the planning, process, and evaluation of translating patient-reported outcome systems into routine clinical care has been strongly recommended [37]. A notable strength of this study was the use of the CFIR to inform the interview guide and categorize the determinants of the implementation of an ePSM. This will assist in the selection of implementation strategies for the implementation of REACH. Another strength of this study was the large number of participants interviewed, the representation of diverse clinic staff roles (e.g., leadership and management, physicians, nursing, allied health), and the representation of patients and clinical staff from each centre and disease site involved in the initial implementation of REACH. These four cancer centres vary in terms of size, resources, and patient diversity, which provide a more generalizable understanding of the barriers and facilitators to successful implementation.

This study also has limitations. First, the findings from this study may be more applicable to a remote ePSM system similar to REACH rather than any ePSM system. REACH is completely remote and automated, meaning that patients will complete their symptom screening questions on their own time outside of clinic visits. Following the completion of the surveillance questions, REACH will automatically provide patients with self-management resources, community programs, or suggested referrals to rehabilitation professionals and programs based on the responses given. This may differ from other ePSM systems that may ask patients to complete symptom screening questions in-clinic and generate a summary report for clinicians with recommended clinical actions and referrals. Second, an updated version of the CFIR was published after the qualitative data were analyzed for this study. The updated CFIR includes refinements to existing constructs and the addition of new constructs [57]. Future studies should explore how these new constructs, such as the sub-construct Information Technology Infrastructure within the inner setting (i.e., technological systems for telecommunication, electronic documentation, and data storage, management, reporting, and analysis), and the construct Local Conditions within the outer setting (i.e., economic, environmental, political, and/or technological conditions that enable implementation) influence the implementation of an ePSM. Lastly, participant characteristics were not collected as part of this pre-implementation examination; thus, these findings may not be representative of a range of patient and provider views. For example, we see that HCP were largely from the two larger cancer centres; these findings may underrepresent views of those working in rural and less-resourced settings.

## Conclusions

This study identified implementation barriers and facilitators that can be used to develop a tailored implementation plan for an ePSM designed to address physical impairments in people living with and beyond cancer across all five CFIR domains. The adaptability, complexity, and perceived relative advantage of an ePSM emerged as key at the intervention-level, along with system knowledge at the individual level. At the inner setting level, major determinants were the compatibility of an ePSM with clinical workflows and the level of resources required, while the need to meet the needs of patients and availability of rehabilitation supports were important considerations from the outer setting. Engaging various stakeholders was expected to be a key step in the implementation process. The findings from this study demonstrate the importance of obtaining input from key stakeholders before the implementation of an ePSM. A pragmatic implementation study is underway to evaluate the implementation of the REACH system, which will provide insight into whether or not identified barriers were successfully mitigated through appropriately tailored implementation strategies.

### Electronic supplementary material

Below is the link to the electronic supplementary material.

**Supplementary Material 1:** Focus Group Topic Guide - Patient Participants

**Supplementary Material 2:** Focus Group Topic Guide - Staff Participants

## Data Availability

The dataset used and analysed during the current study are available from the corresponding author on reasonable request.

## Abbreviations

CFIR: Consolidated Framework for Implementation Research
ePROs: Electronic patient reported outcomes
ePSM: Electronic Prospective Surveillance Model
ERIC: Expert Recommendations for Implementing Change
KTA: Knowledge-to-Action
PFAC: Patient and Family Advisory Committee
PSM: Prospective Surveillance Model
SRQR: Standards for Reporting Qualitative Research
