# Supplementary material for: A pre-implementation examination of barriers and facilitators of an electronic prospective surveillance model for cancer rehabilitation: a qualitative study
Source: BMC Health Serv Res. 2024 Jan 4;24:17. doi: 10.1186/s12913-023-10445-3 (PMC10768357; doi:10.1186/s12913-023-10445-3)
Supplement: Supplementary file 1 — Supplementary Material 1: Focus Group Topic Guide - Patient Participants [file 12913_2023_10445_MOESM1_ESM.docx]

**Implementing an Electronic Prospective Surveillance Model for Cancer Rehabilitation**

**Focus Group Topic Guide – Patient Participants**

**Preamble**

Thank you for taking the time to meet with us. As you may recall, we are preparing to implement an online system called **REACH.** This system will screen patients for physical impairments throughout their care and connect them with rehabilitation services based on need. We would like to understand how to facilitate the registration and use of the system. Your perspectives will help us develop a plan to support implementation success.

This meeting will be audio recorded to ensure your experiences are being accurately represented. Your responses will remain confidential, and your personal information will not be shared with anyone. Please feel free to skip over any questions that you do not feel comfortable answering.

Do you have any questions before we begin?

[No] Wonderful. I will begin recording if that is okay with you?

**Questions**

1. Let’s start with a brief round of introductions. Please tell us your name, where you are joining us from today, and, very briefly, where you are in your cancer journey.

Before we begin with questions, I would like to present a brief overview of the system and its design.

1. Do you have any questions about the system? *[Intervention Characteristics]*
   1. Consider how complicated or easy the system is to use. *[Intervention Characteristics > Complexity]*
   2. Consider how the system’s goals and benefits compares to existing interventions/systems. *[Intervention Characteristics > Relative Advantage]*
   3. Consider features that should be changed or added. *[Intervention Characteristics > Adaptability]*

Next, I would like to understand how the implementation of the system could fit within the cancer pathway.

1. When would be a suitable time to have the system introduced to patients? For instance, this could be an initial visit with the oncology team, a follow up visit before treatment, right after beginning treatment, etc. [*Process > Engaging > Intervention Participants]*
2. Who might be the person (or people) to introduce and explain the ePSM to patients? Would it be the surgical/medical/radiation oncologists, nurses (which nurse in which role), radiation therapists, and/or administrative staff? [*Process > Engaging > Key Stakeholders]*
3. What type of materials will be helpful to ensure patients understand the purpose of REACH, how to register, and/or use the system? Some examples may include pamphlets provided to you or posted in the clinic, instructional videos, a website, and/or an email contact. *[Process > Engaging > Intervention Participants]*
4. What supports or materials will be helpful to ensure patients complete their questionnaires on REACH throughout their care? Some examples may include alerts on the app, automated emails, different ways of viewing your scores over time, and/or discussions about your scores with your oncology team. *[Process > Engaging > Intervention Participants]*

Is there anything else that you would like to share related to the implementation of the system?

Thank you for taking the time to participate in this interview.
