# Supplementary material for: A pre-implementation examination of barriers and facilitators of an electronic prospective surveillance model for cancer rehabilitation: a qualitative study
Source: BMC Health Serv Res. 2024 Jan 4;24:17. doi: 10.1186/s12913-023-10445-3 (PMC10768357; doi:10.1186/s12913-023-10445-3)
Supplement: Supplementary file 2 — Supplementary Material 2: Focus Group Topic Guide - Staff Participants [file 12913_2023_10445_MOESM2_ESM.docx]

**Implementing an Electronic Prospective Surveillance Model for Cancer Rehabilitation**

**Focus Group Topic Guide – Staff Participants**

**Preamble**

Thank you for taking the time to meet with us. As you may recall, we are preparing to implement an online system called **REACH.** This system will screen patients for physical impairments throughout their care and connect them with self-management and rehabilitation services based on need. The study is intended to inform the development and selection of strategies to facilitate implementation and sustainment of the system. Throughout the interview I will be asking questions about your current workflow, clinic structure, thoughts on the benefits or drawbacks of using the system, and your thoughts on the clinic readiness for implementing the system. I will also ask you to discuss potential strategies that may be used to facilitate implementation of the system at your site. Please be as forthright as possible as our team is fully invested in making the system as useful as possible, even if it means making substantial changes to the system itself. Your perspectives will help us develop a plan to support implementation success.

The interview is expected to last approximately 60 minutes in length and will be audio recorded to ensure your experiences are being accurately represented. Your responses will remain confidential, and your personal information will not be shared with anyone. Please feel free to skip over any questions that you do not feel comfortable answering.

Do you have any questions before we begin?

[No] Wonderful. I will begin recording if that is okay with you?

**Questions**

1. Let’s start with a brief round of introductions. Please describe your role within your organization/clinic.

Before we begin with questions, I would like to present a brief overview of the system and its design.

1. Do you have any questions about the system? *[Intervention Characteristics]*
   1. Consider how complicated or easy the system is to use. *[Intervention Characteristics > Complexity]*
   2. Consider how the system’s goals and benefits compares to existing interventions/systems. *[Intervention Characteristics > Relative Advantage]*
   3. Consider features that should be changed or added. *[Intervention Characteristics > Adaptability]*

Next, I would like to understand the existing work processes and practices in your setting.

1. Do patients currently complete any PROs as part of routine care in your setting? If so, is this done electronically? What type of measures do they complete? *[Inner Setting > Implementation Climate]*
   1. To what extent might this system take a back seat to other initiatives? *[Inner Setting > Implementation Climate > Relative Priority]*
2. How well does the system (the symptoms screened and resources offered) fit with existing work processes and practices in your setting? *[Inner Setting > Implementation Climate > Compatibility]*
   1. Will the system replace or compliment a current program or process? How?
   2. What are likely issues or complications that may arise?

Next, I would like to understand how the implementation of the system could fit within these existing processes.

1. When would be a suitable time to have the system introduced to patients? For instance, this could be an initial visit with the oncology team, a follow up visit before treatment, etc. [*Process > Engaging > Intervention Participants]*
2. Who might be the person (or people) to introduce and explain the system to patients? Would it be the surgical/medical/radiation oncologists, nurses (which nurse in which role), radiation therapists, and/or administrative staff? [*Process > Engaging > Key Stakeholders]*
3. What kinds of information and materials about the system should be planned for both providers and patients in your setting? *[Inner Setting > Readiness for Implementation > Access to Knowledge and Information & Process > Engaging > Key Stakeholders]*
   1. What should be the communication or education strategy for getting the word out about the system to patients and staff? What materials/modes/venues should be used? For example, e-bulletin boards, emails, brochures?
   2. For providers, what steps should be taken to encourage them to offer the system to patients?

Given everything we’ve discussed, I’d like to hear your feedback on when we should implement the system in your setting.

1. When might be a suitable time to implement the system? Why? What would help get you ready to implement the system? *[Inner Setting > Readiness for Implementation]*
2. Are there any other key individuals to get on board with the implementation of the system? *[Process > Engaging]*
   1. Who should help lead implementation of the system at your setting? What role do they have in the setting?

Is there anything else that you would like to share related to the implementation of the system?

Thank you for taking the time to participate in this interview.
